# Childhood Mortality Due to Unintentional Injuries in Japan, 2000–2009

**DOI:** 10.3390/ijerph10020528

**Published:** 2013-01-30

**Authors:** Hideaki Sekii, Tadahiro Ohtsu, Takako Shirasawa, Hirotaka Ochiai, Takaya Shimizu, Akatsuki Kokaze

**Affiliations:** Department of Public Health, Showa University School of Medicine, 1-5-8 Hatanodai, Shinagawa-ku, Tokyo 142-8555, Japan; E-Mails: hideaki.sekii@nifty.ne.jp (H.S.); shirasawa@med.showa-u.ac.jp (T.S.); h-ochiai@med.showa-u.ac.jp (H.O.); takaya@shimizu.gr.jp (T.S.); akokaze@med.showa-u.ac.jp (A.K.)

**Keywords:** unintentional injury, childhood mortality, cause of death, Japan

## Abstract

This study examined deaths due to unintentional injuries among children in Japan to identify the age groups and sexes at most risk, and the types of injuries, so that effective forms of targeted intervention can be devised. Among children aged 0–14 years, deaths whose underlying causes had been classified under code V01-X59 of the ICD-10 were defined as deaths of children caused by unintentional injuries. Using data from the *Vital Statistics* 2000–2009 for analysis, we examined the changes in mortality and trends in terms of sex, age, and cause of death. Mortality decreased by 46.2%, from 933 in 2000 to 502 in 2009. The mortality rate among children aged 1–4 years decreased by almost half. The total number of deaths during this decade was 7,362 (boys: 4,690, girls: 2,672). Among the causes of death, the majority were due to “transport accidents”, followed by “other accidental threats to breathing”, and “accidental drowning and submersion”. The characteristics observed in terms of sex, age, and cause of death—that is, deaths from suffocation among infants aged less than 1 year, drowning deaths among boys, and transport accidents involving pedestrians and cyclists—must be addressed as targets for future intervention.

## 1. Introduction

Injuries are the leading cause of death and long-term disability in childhood, and contribute significantly to healthcare costs in most of the developed countries [1,2,3]. In 2005, the World Health Organization (WHO) issued *Child and adolescent injury prevention: A WHO plan of action 2006–2015* [4]. Its introduction stated that more than 95% of all injury-related deaths worldwide among children and adolescents occurred in low- and middle-income countries, but that even in high-income countries, injuries were still a major cause of death among children and adolescents, accounting for about 40% of all deaths among those aged between 1 and 18 years. In addition, the *National Action Plan for Child Injury Prevention*, which was announced by the Centers for Disease Control and Prevention (CDC) in 2012, stated that in 2009 alone, 7,962 children (aged 1–19 years) in the USA had died from unintentional injuries, accounting for nearly 37% of all post-infancy deaths among children [5].

Similarly, in Japan, unintentional injuries have been the leading cause of death among children aged 1 year or older since 1960 [6,7,8]. The prevention of unintentional injuries in childhood is thus one of the nation’s most important issues related to juvenile health. In November 2000, the then Ministry of Health and Welfare launched a national campaign “*Healthy Family 21*”, which was intended to offer directions for addressing maternal and child health at the beginning of the 21st century [9]. One of the main issues of this campaign was environmental improvement to maintain or raise healthcare standards for children. The rate of mortality attributable to unintentional injuries was considered to be one of the key indices of child healthcare, and a goal was set to reduce the rate by half before 2010. For reference, the mortality rates of infants (under 1 year) and young children (1–4 years) in Japan in 2000 were 3.2 (per thousand births) and 30.6 (per hundred thousand children), respectively [10].

Hence, the present study uses data from the national *Vital Statistics* from the decade beginning in 2000, when “*Healthy Family 21*” was launched. We examined the sexes and ages of children in Japan who had died of unintentional injuries, together with the specific cause of death, in order to identify the age groups and sexes at risk, and the most common types of injuries, so as to assist the development of effective forms of targeted intervention.

## 2. Methods

### 2.1. Definition of Deaths among Children Due to Unintentional Injuries, and Data Used for Analyses

*The International Statistical Classification of Diseases and Related Health Problems Tenth Revision* (ICD-10) has been used to calculate mortality statistics in Japan since 1995 [11]. We defined deaths among children aged 0–14 years whose underlying causes of death [12] had been classified under any of the ICD-10 3-digit codes V01–X59 (V01–V98, W00–W99, and X00–X59) [13] as being attributable to unintentional injuries. Japan’s Ministry of Health, Labour and Welfare has been performing death-cause coding according to the ICD, using data from death certificates recorded by physicians, for more than a hundred years since the introduction of the ICD-1 in 1900 [14]. For the analyses, we used *Vital Statistics* data for a period of 10 years from 2000, when “*Healthy Family 21*” was launched [15].

### 2.2. Data Analysis

First, a bar graph of the sex-based childhood deaths for each year was created to observe the annual changes. Then the mortality rates (total number of boys and girls per hundred thousand children) of children aged under 1 year, and those aged 1–4 years, 5–9 years, and 10–14 years in 2009 were calculated on the basis of the estimated population [16]. The proportions of deaths caused by unintentional injuries among the total number of deaths among children aged less than 1 year and those aged 1–14 years in 2000 and 2009 were also calculated.

Second, we aggregated the numbers of deaths by age class from 2000 to 2009 separately for boys and girls and calculated the male-to-female proportion ratios (PRs) and their 95% confidence intervals (95% CIs). For adjustment of the PR for age, Mantel-Haenszel stratified analysis was used.

Third, we compared the degrees of decrease, on the basis of age class, in the deaths of children caused by unintentional injuries from 2000 to 2009, separately for boys and girls.

Fourth, we aggregated the numbers of deaths by age class on the basis of major causes from 2000 to 2009, separately for boys and girls, and calculated the male-to-female PR for each cause of death and its 95% CI.

Fifth, we compared the degrees of decrease, on the basis of cause of death, in the deaths of children caused by unintentional injuries from 2000 to 2009, separately for boys and girls.

Finally, we examined the major causes of death—“transport accidents” (ICD-10 code: V01–V98), “accidental drowning and submersion” (W65–W74), and “other accidental threats to breathing” (W75-W84)—among the deaths from unintentional injuries in 2009, using 3-digit ICD-10 codes.

Epi Info Version 3.3.2 was used for statistical analyses.

### 2.3. Ethical Aspects

Since the present study used only published government data, approval from an ethics committee was not required.

## 3. Results

**Figure 1:**
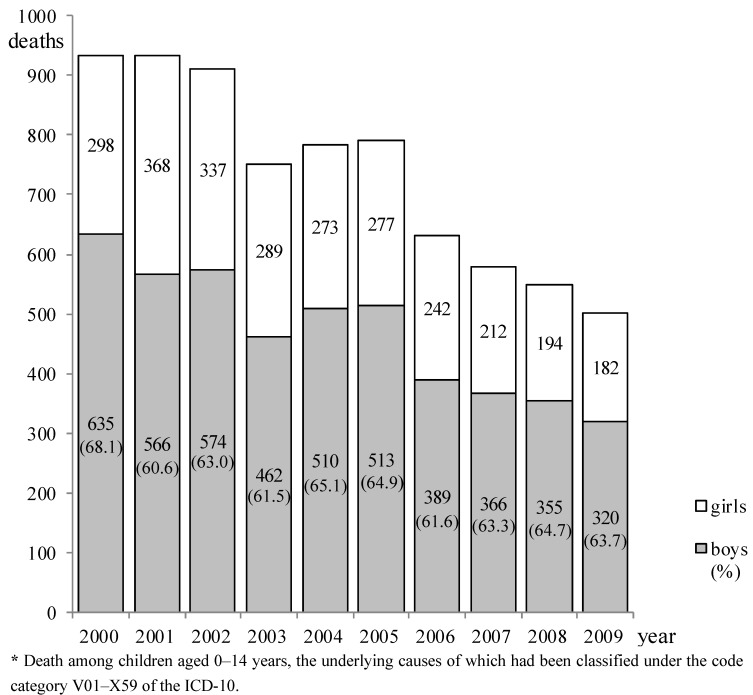
Annual changes in childhood deaths due to unintentional injuries * by sex, 2000–2009.

The annual changes in mortality are indicated in Figure 1. Mortality tended to decrease during the decade, from a total of 933 children (boys: 635, girls: 298) in 2000 to 502 (boys: 320, girls: 182) in 2009. The mortality rates (total number of boys and girls per hundred thousand children) among children aged under 1 year, and those aged 1–4 years, 5–9 years, and 10–14 years in 2009 were 11.6, 3.5, 2.4, and 1.6, respectively.

The proportions of deaths resulting from unintentional injuries among the total number of deaths of infants younger than 1 year and children aged 1–14 years were 5.7% (217 of 3,830) and 24.5% (716 of 2,921), respectively, in 2000, and 4.9% (124 in 2,556) and 19.6% (378 in 1,925), respectively, in 2009.

Sex-based mortalities by age class are shown in Table 1. A total of 7,362 children (4,690 boys and 2,672 girls) died during the decade, among whom infants aged under 1 year and children aged 1–4 years accounted for 54.1% (1,615 + 2,371 deaths, respectively, out of the total 7,362). The male-to-female PRs were significantly high for all age classes, and increased along with age. The age-adjusted male-to-female PR was 1.76 (95% CI: 1.66–1.86) for children aged 0–14 years.

**Table 1:** Sex-based childhood deaths due to unintentional injuries * by age class, aggregated from 2000 to 2009.

Age (year)	Boys (%)	Girls (%)	Total (%)	The male-to female proportion ratio (95% CI)
<1	946 (20.2)	669 (25.0)	1,615 (21.9)	1.41 (1.25–1.60)
1–4	1,478 (31.5)	893 (33.4)	2,371 (32.2)	1.66 (1.49–1.83)
5–9	1,331 (28.4)	679 ()25.4	2,010 (27.3)	1.96 (1.75–2.19)
10–14	935 (19.9)	431 (16.1)	1,336 (18.6)	2.17 (1.89–2.49)
0–14	4,690 (100)	2,672 (100)	7,362 (100)	1.76 (1.66–1.86) **

* Death among children aged 0–14 years, the underlying causes of which had been classified under the code category V01–X59 of the ICD-10. ** Mantel-Haenszel stratified analysis was used for age adjustment.

The sex-based comparison of the number of deaths from unintentional injuries by age class in 2000 and 2009 is shown in Table 2, which indicates the decrease in terms of both the numbers and ratios of deaths between the two years. The decrease in deaths was greater for boys than for girls; the number of boys’ deaths had decreased by almost half. In terms of the number of deaths, the decrease was largest for boys aged 1–4 years (the ratio of deaths in 2009 to those in 2000: 0.431), and the decreases in the number of deaths among girls aged 1–4 years and 5–9 years were almost the same (ratios: 0.588 and 0.512, respectively), these decreases being larger than those for other age classes. Among infants aged less than 1 year, the ratios of deaths in 2009 to those in 2000 for boys and girls (0.529 and 0.646, respectively) were both larger than those for the corresponding totals (*i.e.*, the decreases were smaller).

**Table 2:** Sex-based numbers of childhood deaths due to unintentional injuries * by age class, comparison between the years 2000 and 2009.

Age (year)		2000	2009	Numbers of decrease	Ratio **
*Boys*					
	<1	138	73	65	0.529
	1–4	211	91	120	0.431
	5–9	158	95	63	0.601
	10–14	128	61	67	0.477
	Total	635	320	315	0.504
*Girls*					
	<1	79	51	28	0.646
	1–4	97	57	40	0.588
	5–9	84	43	41	0.512
	10–14	38	31	7	0.812
	Total	298	182	116	0.611
* Deaths among children aged 0–14 years, the underlying causes of which had been classified under the code category V01–X59 of the ICD-10. ** Ratio of the value in 2009 to that in 2000.					

Sex-based numbers of deaths attributable to major causes by age class are shown in Table 3. With regard to deaths among boys, the number attributable to “transport accidents” (V01–V98) was the largest, followed by “other accidental threats to breathing” (W75–W84) and “accidental drowning and submersion” (W65–W74), with a slim margin between the latter two. Among girls, the number of deaths attributable to “transport accidents” was the largest, followed by “other accidental threats to breathing” and “accidental drowning and submersion”. These three causes of death accounted for approximately 80% (boys: 3,789 of 4,690, girls: 2,112 of 2,672) of deaths due to unintentional injuries. Adding “exposure to smoke, fire, and flames” (X00–X09) and “falls” (W00–W17) to the above three causes accounted for almost 95% (boys: 94.8% (4,447), girls: 94.3% (2,521)) of all deaths resulting from unintentional injuries during the 10-year period. With regard to the proportion of deaths by age class, “transport accidents” accounted for the largest proportion among both boys and girls aged 5–9 years, followed by those aged 1–4 years. With regard to deaths from “other accidental threats to breathing”, infants aged less than 1 year accounted for around 65% of the total. With regard to “accidental drowning and submersion”, the proportions of deaths among both boys and girls aged 1–4 years were the largest. The male-to-female PRs were significantly high for any of the causes of death. The PR for “accidental drowning and submersion” was particularly high at 2.36 (95% CI: 2.06–2.70).

The sex-based comparison of the numbers of deaths from unintentional injuries by cause of death in 2000 and 2009 is shown in Table 4, which indicates the decrease in terms of both the numbers and ratios of deaths between the two years. 

**Table 3:** Sex-based numbers of childhood deaths due to major unintentional injuries * by age class, aggregated from 2000 to 2009.

ICD-10 code		Cause of death	＜1 year	1–4 year	5–9 year	10–14 year	Total	Ratio (95% CI) ***
*Boys*								
	V01–V98	Transport accidents	73 (4.3) **	499 (29.5)	676 (40.0)	442 (26.2)	1,690 (100)	1.78 (1.61–1.96)
	W00–W17	Falls	47 (14.6)	149 (46.4)	52 (16.2)	73 (22.7)	321 (100)	2.07 (1.63–2.62)
	W65–W74	Accidental drowning and submersion	51 (5.0)	391 (38.3)	366 (35.8)	213 (20.9)	1,021 (100)	2.36 (2.06–2.70)
	W75–W84	Other accidental threats to breathing	690 (64.0)	239 (22.2)	77 (7.1)	72 (6.7)	1,078 (100)	1.48 (1.32–1.66)
	X00–X09	Exposure to smoke, fire and flames	18 (5.3)	134 (39.8)	114 (33.8)	71 (21.1)	337 (100)	1.33 (1.08–1.63)
	Total		879	1,412	1,285	871	4,447	–
*Girls*								
	V01–V98	Transport accidents	58 (6.1) **	321 (33.8)	355 (37.4)	216 (22.7)	950 (100)	Described in the preceding corresponding cell
	W00–W17	Falls	23 (14.8)	77 (49.7)	25 (16.1)	30 (19.4)	155 (100)	
	W65–W74	Accidental drowning and submersion	46 (10.6)	187 (43.2)	130 (30.0)	70 (16.2)	433 (100)	
	W75–W84	Other accidental threats to breathing	486 (66.7)	161 (22.1)	47 (6.4)	35 (4.8)	729 (100)	Ditto
	X00–X09	Exposure to smoke, fire and flames	18 (7.1)	90 (35.4)	84 (33.1)	62 (24.4)	254 (100)	
	Total		631	836	641	413	2,521	
* Deaths among children aged 0–14 years, the underlying causes of which had been classified under the code category V01–X59 of the ICD-10. ** The value in parentheses, adjacent to each value, denotes the proportion (%) of deaths due to the corresponding cause of death by age class. *** The male-to-female proportion ratio.								

The decrease in the number of deaths attributable to “transport accidents” was the largest for boys, followed by deaths due to “other accidental threats to breathing”. In girls, the decrease in the number of deaths attributable to “other accidental threats to breathing” was the largest, followed by that of deaths due to “transport accidents”. In boys, the ratio of deaths from “accidental drowning and submersion” in 2009 to those in 2000 was the largest (*i.e.*, the decrease in incidence was the smallest), whereas in girls, the ratio of deaths due to “accidental drowning and submersion” in the two years was smaller (*i.e.*, the decrease in incidence was larger), next to that for “other accidental threats to breathing”. The ratio of the proportion of deaths attributable to “transport accidents” relative to all deaths caused by unintentional injuries in 2002 (40.2%; 366 out of 911), when it was highest, and that in 2009 (29.7%; 149 out of 502), when it was lowest, was 0.74 (95% CI: 0.59–0.93). Therefore, a significant decrease was evident. With regard to girls aged 10–14 years, among whom was observed the smallest decrease in the number of deaths due to unintentional injuries from 2000 (38) to 2009 (31) (Table 2), changes in the number of children who died due to various causes revealed increases in the number of deaths caused by “falls” and “accidental drowning and submersion”, with a total of 9 additional deaths.

**Table 4:** Sex-based numbers of childhood deaths due to unintentional injuries * by cause of death, comparison between the years 2000 and 2009.

ICD-10 code		Cause of death	2000	2009	Numbers of decrease	Ratio **
*Boys*						
	V01–V98	Transport accidents	227	87	140	0.383
	W00–W17	Falls	62	28	34	0.452
	W65–W74	Accidental drowning and submersion	127	91	36	0.717
	W75–W84	Other accidental threats to breathing	146	77	69	0.527
	X00–X09	Exposure to smoke, fire and flames	45	14	31	0.311
*Girls*						
	V01–V98	Transport accidents	98	62	36	0.633
	W00–W17	Falls	15	14	1	0.933
	W65–W74	Accidental drowning and submersion	53	32	21	0.604
	W75–W84	Other accidental threats to breathing	89	50	39	0.562
	X00–X09	Exposure to smoke, fire and flames	24	15	9	0.625

* Deaths among children aged 0–14 years, the underlying causes of which had been classified under the code category V01–X59 of the ICD-10. ** Ratio of the value in 2009 to that in 2000.

The breakdown of the major causes of 502 deaths from unintentional injuries in 2009 was as follows: Among 149 deaths due to “transport accidents”, “pedestrians injured in transport accidents” (V01–V09) accounted for 50.3% (75 deaths), followed by “pedal cyclists injured in transport accidents” (V10–V19) at 26.2% (39 deaths), and “car occupants injured in transport accidents” (V40–V49) at 19.5% (29 deaths). Among 127 deaths due to “other accidental threats to breathing”, 29 deaths due to “inhalation of gastric contents” (W78) and 25 deaths due to “inhalation and ingestion of food causing obstruction of respiratory tract” (W79) together accounted for 42.5%, followed by 39 deaths (30.7%) due to “accidental suffocation and strangulation in bed” (W75). Among 123 deaths due to “accidental drowning and submersion”, 50 deaths due to both “drowning and submersion while in bath tub” (W65) and “drowning and submersion following fall into bath tub” (W66) accounted for 40.7%, and 39 deaths due to both “drowning and submersion while in natural water” (W69) and “drowning and submersion following fall into natural water” (W70) accounted for 31.7%.

## 4. Discussion

This study, based on an analysis of data from the national *Vital Statistics*, clarified changes in patterns of death due to unintentional injuries among children in Japan over the decade preceding the Great East Japan Earthquake, which occurred on March 11, 2011 [17], and their characteristics in terms of sex, age, and cause of death. We believe the basic data we have obtained will be valuable for revealing the age groups and sexes most at risk, and the types of injuries involved, so that useful forms of targeted intervention can be devised.

The total number of deaths among boys and girls decreased from 933 in 2000 to 502 in 2009 (a decline of 46.2%) (Figure 1). Such a decrease in mortality during the decade can be considered a substantial improvement, even after taking into account the fall in the number of live births in Japan during this period (approximately 10%) [15]. In 2000, when “*Healthy Family 21*” was launched, the rates of mortality (per hundred thousand children) resulting from unintentional injuries were 18.2, 6.6, 4.0, and 2.6 for children aged under 1 year, and those aged 1–4 years, 5–9 years, and 10–14 years, respectively [10]. In comparison with those values, the mortality rates for 2009 obtained in the present study were substantially lower, particularly among children aged 1–4 years (6.6 to 3.5, a decrease of 47.0%), the decrease almost attaining the 50% goal by 2010 set by this campaign. (This deadline was later extended for 4 years, and the current deadline is 2014 [9].) This achievement is significant because the rate of mortality among children aged 1–4 years attributable to unintentional injuries in Japan was higher than the average mortality rate for developed countries as a whole [6]. At the launch of Healthy Family 21, as a task for the government and organizations concerned, a goal was set that all municipalities would implement measures to prevent injuries involving infants and young children, by taking advantage of medical checkups for them (the target percentage of the municipalities performing such measures has currently been lowered from 100% to 50–55%) [10].

The proportion of deaths caused by unintentional injuries among total deaths in infants aged less than 1 year remained at around the 5% level, and that for children aged 1–14 years showed only a modest decrease from 24.5% in 2000 to 19.6% in 2009. These data suggest that unintentional injuries remain an important cause of death in children in Japan. The proportion of such deaths among children aged 1–14 years in Scotland for the whole 5-year period between 2002 and 2006 was reported to be 20.6% (138 out of a total of 669 deaths) [18], being similar to our present result. By contrast, the proportion of such deaths among children aged 1–14 years in the USA in 2009 was 31.2% (3,155 out of a total of 10,101 deaths) [5], which was higher than both the proportions reported in this study and the proportion in the previous study conducted in Scotland [18].

In Japan, the total number of deaths due to unintentional injuries among boys and girls aged 0–14 years during the 10 years from 2000 to 2009 was 7,362, and more than half were in infants aged under 1 year and children aged 1–4 years (Table 1). As mentioned above, the rate of decrease in deaths was greater among children aged 1–4 years than among children in other age classes. In addition, the ratios of the number of deaths among boys and girls aged less than 1 year in 2009 to those in 2000 were larger than the values for the respective totals (Table 2). Therefore, infants aged less than 1 year must be targeted to reduce the number of such deaths occurring among them.

The male-to-female PR for children aged under 1 year was significantly higher, at 1.41 (95% CI: 1.25–1.60), and the value increased with age (Table 1). This phenomenon supports the statement in the WHO report that the gender gap in injury-related deaths increases with age in most regions and countries. The same report stated that the male-to-female difference in injury-related death rates (including intentional injuries) tends to be more pronounced in higher-income nations, where the injury-related death rate among males is 50% higher than that among females [4]. Furthermore, a study using injury mortality data for children aged 0–14 years between 1982 and 2006 in Scotland indicated that the age-adjusted male-to-female ratio was 1.70 (95% CI: 1.56–1.86) [19]. The age-adjusted male-to-female PR obtained in the present study was 1.76 (95% CI: 1.66–1.86) for children aged 0–14 years. This particularly large gender gap, similar to the result obtained in Scotland, must be the focus when examining measures to prevent deaths among children due to unintentional injuries in Japan.

According to a review of pediatric unintentional injury, boys suffered injuries more often than girls did, an indisputable finding that has been consistently reported historically, cross-culturally, and developmentally [20]. In the present study, the male-to-female PRs were calculated in terms of cause of death, and significant increases in all proportion ratios were recognized (Table 3), in accord with the above statement. It is noteworthy that in the WHO report, which included cause- and sex-specific data on injury-related deaths among children under the age of 15 years in 2002, the male-to-female ratios of deaths caused by road traffic and by drowning were almost the same (1.56 and 1.63, respectively) [4], whereas the results obtained in the present study indicated a higher male-to-female ratio of deaths caused by accidental drowning and submersion (2.36) than by transport accidents (1.78). In addition, the ratio of the number of boys’ deaths due to accidental drowning and submersion in 2009 relative to that in 2000 was the largest (Table 4), an issue that requires immediate attention.

Deaths caused by transport accidents accounted for the largest proportion (35.9% (a total of 2,640, with 1,690 boys and 950 girls), Table 3) of deaths attributable to unintentional injuries during the decade beginning in 2000. It was reported that road traffic injuries accounted for a significant proportion of unintentional injury deaths in all WHO regions, and that road traffic injuries were responsible for the largest proportion of unintentional injury deaths (33%) in 2004, although this value was for individuals in all age groups [21]. This value is similar to that for children in Japan. However, the comparison between the years 2000 and 2009 indicated that the decrease in the number of boys’ deaths caused by transport accidents was the largest, and that the decrease in the number of girls’ deaths caused by transport accidents was the second largest, following deaths caused by other accidental threats to breathing (Table 4). In addition, there was a significant decrease in the proportions of deaths caused by transport accidents among all deaths due to unintentional injuries: from 40.2% in 2002 to 29.7% in 2009. This decline could have been attributable to the substantial reduction in the number of fatal accidents in Japan caused by drunk driving, following the introduction of strong penalties after the amendment of the Road Traffic Act in June 2002 [22].

With regard to the number of deaths attributable to major causes by age class aggregated from 2000 to 2009, distinct differences in age distribution were observed. For example, infants less than 1 year accounted for around 65% of the number of deaths due to other accidental threats to breathing (Table 3). This suggests the importance of developing preventive measures against unintentional injuries based on the growth and development stages of children. A study of injury mortality among children younger than 15 years conducted between 1979 and 2002 in Canada reported that for infants aged under 1 year, suffocation was the most common cause of injury-related death [2]. In addition, another report from the USA indicated that drowning was the leading cause of injury-related death in children aged 1 to 2 years [23]. Furthermore, a CDC document pointed out that more than three-quarters of deaths due to injury among children below 1 year of age were due to suffocation [5]. These findings support the present results.

We examined the characteristics of deaths attributable to transport accidents in 2009 using the 3-digit codes of the ICD-10, and the proportions of deaths among pedestrians, pedal cyclists, and car occupants were 50.3%, 26.2%, and 19.5%, respectively. A report published in 2008 by the WHO detailed the proportions of road traffic deaths among children under the age of 15 years according to the type of road user in selected OECD countries. On the basis of this report, we found that only 4 of the 19 countries covered (the Netherlands, the Republic of Korea, Switzerland, and the United Kingdom) had proportions of car passenger deaths similar to that found in the present study. In other countries, the proportions of deaths among car passengers were higher [24]. This could be interpreted as indicating that the safety of juvenile car passengers is relatively higher in Japan. However, the risk of death caused by accidents to pedestrians and cyclists may be higher. Therefore, continuous efforts are required, particularly in ensuring the safety of pedestrians and cyclists. It has been reported that the bicycle safety helmet legislation in California was associated with an 18.2% reduction in the proportion of traumatic brain injuries among injured youth bicyclists aged 17 years and younger [25].

The decrease in the number of deaths due to other accidental threats to breathing was large among both boys and girls (Table 4). A breakdown of this category for 2009 indicated that the total proportion of deaths due to “inhalation of gastric contents” and “inhalation and ingestion of food causing obstruction of respiratory tract” was 42.5%, followed by deaths due to accidents in bed (30.7%). Hence, to prevent deaths from suffocation, children’s body posture must be monitored during and after meals, and a safe sleeping environment must be maintained [26]. The breakdown of deaths due to accidental drowning and submersion in 2009 indicated that 40.7% had taken place in a bath tub. In “*Healthy Family 21*”, the proportion of households with infants and young children that implemented countermeasures against children opening the bathroom door by themselves was set as a behavioral indicator [10]. However, it would be more preferable to devise complex safety structures to ensure that infants and young children are not at risk of injury even if they open the bathroom door by themselves.

## 5. Study Limitations

This study had some limitations. First, it analyzed data only for children who had suffered deaths due to unintentional injuries, and not all children who suffered unintentional injuries. According to a survey of 96,359 children born between April 1985 and March 1988 in Canada until the age of 9 years, nearly 84% of them received care for an injury of some sort during the study period, and in any given year, approximately 21% of the studied population suffered at least one injury [27]. It has been said that “Deaths represent only the proverbial “tip of the iceberg” of the true burden of unintentional injuries” [21], and it must be noted that the respective ratio of fatal injuries, injuries leading to hospitalization, and injuries requiring a visit to a clinic among children aged 1–4 years in Japan has been estimated to be 1:65:5,850 [6]. Second, the data used for the present analyses were only sex, age at death, year of death, and the ICD-10 codes for underlying causes of death. Details of the circumstances of individual accidents were not clear. In addition, other factors possibly relevant to deaths caused by unintentional injuries, for example the characteristics of individual home environments such as the parental attitude toward child-rearing, the presence of siblings, economic context, and the regional conditions of residence such as access to medical facilities, were not considered. The results of a meta-analysis on parenting interventions and the prevention of unintentional injuries during childhood showed that intervention-arm families had a significantly lower risk of injury [28]. Moreover, in Japan, a population-based birth cohort study conducted for 42,144 individuals concluded that paternal involvement in childcare might be a useful predictive indicator of childhood injury [8]. Third, the degree of misclassification of death causes in the *Vital Statistics* data used in our study is not known. For verification of such misclassification, primary data (death certificates) should be obtained, but permission for this is granted only after rigorous and prolonged screening from the administrative authorities, and hence such verification is very difficult. Finally, we cannot determine the reasons for the decrease in childhood mortality due to unintentional injuries on the basis of our study results. A CDC document stated that research to improve prevention efforts is needed at the following three levels: (1) foundational research (how injuries occur), (2) evaluative research (what works and what does not work to prevent injuries), and (3) translational research (how to put proven injury prevention strategies into action throughout the nation) [5]. According to this categorization, the present study falls into category (1). Therefore, future studies should perform evaluative (2) and translational (3) research.

## 6. Conclusions

The present study examined children’s deaths due to unintentional injuries in Japan, using data from the *Vital Statistics* for the period 2000–2009. Childhood mortality due to unintentional injuries decreased by 46.2%, from 933 in 2000 to 502 in 2009. In particular, the mortality rate among children aged 1–4 years decreased by almost half during this decade.The decrease in mortality can be considered a substantial improvement, even when taking into account the decrease in the number of live births. Because the results of this study indicated trends in certain characteristics, such as sex, age, and cause of death, future targets for intervention must include deaths due to suffocation among infants less than 1 year, drowning deaths among boys, and transport accidents involving pedestrians and cyclists.
